# Editorial: Unraveling vulnerability factors in addiction drug use and potential treatments

**DOI:** 10.3389/fnins.2022.958492

**Published:** 2022-07-29

**Authors:** Jorge Montesinos, Sandra Montagud-Romero, Cristina Núñez

**Affiliations:** ^1^Department of Cellular and Molecular Biology, Centro de Investigaciones Biológicas Margarita Salas (CIB-CSIC), Madrid, Spain; ^2^Department of Psychobiology, Facultad de Psicología, Universitat de València, Valencia, Spain; ^3^Group of Cellular and Molecular Pharmacology, Department of Pharmacology, University of Murcia, Murcia, Spain; ^4^Instituto Murciano de Investigación Biosanitaria (IMIB) - Arrixaca, Murcia, Spain

**Keywords:** substance use disorder (SUD), SUD risk factors, therapeutic approaches against SUD, SUD genetics and epigenetics, animal models for SUD

Addiction is defined as a gradual mental disorder that progresses from social use into a compulsion to take the drug, which results in frequent relapses (Anderson and Hearing, 2019). This progression has been demonstrated to be a consequence of a variety of risk factors, including genetic, epigenetic, sociological, and developmental elements (Anderson and Hearing, 2019). These vulnerabilities are associated with drug-induced neuroadaptations in several brain neurocircuitries, some of them common for all kinds of addictive substances and population while others are drug- or even individual-specific (Everitt and Robbins, 2016). Therefore, only a low percentage of the population that ever uses drugs develop the illness (Everitt and Robbins, 2016). In addition, the relapses in drug intake are recurrent, even after long periods (years) of abstinence, and may be prompted by acute exposure to the drug, drug-associated contexts and cues, stress, the experience of withdrawal symptoms, or exposure to withdrawal-associated cues (Dong et al., 2017). Despite being a main challenge for addiction treatment, the therapeutic approaches along the years have not allowed to diminish the rate of relapses in drug use, that has remained invariable for more than four decades (Dong et al., 2017). Moreover, addiction is not only an increasingly important health problem worldwide with very high socioeconomic impact, but also the use of addictive substances is a vulnerability component for the development of other brain and peripheric illnesses (Klein et al., 2019; Lappin and Sara, 2019; Lowe et al., 2019). Ergo, this Research Topic was aimed to uncover the mechanisms that underlie the progression of the addictive disorder as well as the factors that increase the vulnerability to use addictive drugs in all the stages of this condition in order to contribute to the development of effective pharmacological, psychological, and/or environmental strategies to prevent drug intake and relapses. Some of the studies included in this collection describe how genetics, epigenetics, and sex participate in the development of Substances Use Disorder (SUD). Other articles in this special topic report the influence of lifestyle on a person's vulnerability to use drugs and on the likelihood of relapses, as well as how addictive substances might increase the risk for central and peripheric health problems. Finally, some other studies in this Research Topic provide new inputs in potential therapeutic targets and treatments for SUD.

Genetic and epigenetic factors are known to be relevant in the development of SUDs (Garcia-Perez et al., 2013, 2015; Cadet, 2016; Hamilton and Nestler, 2019). Jordan and Xi reviewed the functional role and relevance of candidate risk genes for the development of SUDs, focusing on the dopamine and glutamate systems and compare available findings between animal models and human reports. For alcohol use disorders (AUDs) genetic risk is around 50%, thus reflecting the importance of a family history of AUD as a risk factor. Therefore, Lundberg et al. examined the heritability of a vulnerability to develop AUDs by comparing the behavioral profiles of several high- vs. low-alcohol consuming selectively bred rat lines and found no common behavioral profile among the high- or the low-alcohol consuming rat lines. Translating this statement into a human population perspective, Blackwood and Cadet provided a short review summarizing relevant genetic and epigenetic factors that contribute to opioid use disorder, with an emphasis on μ-opioid receptor gene. The authors highlight the importance of comprehensive studies comparing individual and group racial differences in order to assess the full behavioral complexity of the spectrum of SUDs.

Gender/sex is an important risk factor for the onset, physical dependence and, likelihood of relapse in drug use. Studies assessing the sex-specific effects and vulnerabilities for SUDs are pivotal in the advancement of the field. In this line, using a fentanyl self-administration rat model of opioid use disorder, Towers et al. reported that female rats self-administered more and showed a higher relapse vulnerability when compared to males, while Cullity et al. examined the sex differences in methamphetamine-conditioned place preference (CPP) in adolescent and adult mice and suggested that the female resistance to form an aversion toward methamphetamine could contribute to the fact that females tend to use methamphetamine at higher rates than males.

Adding to this complexity, the gender differences of the relevance of psychosocial determinants in the onset of AUD was explored by Maxwell et al. When combining the available literature with a machine learning approach, the authors found that social support decreased the levels of negative emotionality, thus protecting against AUD symptom severity in women, while such an effect was not found in men. Indeed, when taking into account both gender and psychological vulnerabilities such as stress or anxiety disorders, complex interactions arise. Employing male and female rats to model the effects of post-traumatic stress disorder-like cues on heroin addiction-like outcomes, Carter et al. found that female rats self-administered more heroin while the reinstatement of drug-seeking behaviors by stress cues was greater in males when compared to females. Moreover, lofexidine, the only FDA-approved non-opioid medication for opioid withdrawal, showed sex-specific impacts on heroin-seeking behaviors following stress in their study. Alcohol use has also been related to cognitive deficits although there is a considerable variability in the animal models used. For that reason, Charlton and Perry reviewed and summarized a variety of factors that may influence the intensity of cognitive decline associated with alcohol consumption such as animal sex (mainly analyzed in males), but also age, doses administered, and time, among others.

Another interesting point analyzed in this Research Topic is the association between addiction and mental health. In this society, there is a high prevalence of co-occurrence of SUD and depression. During the COVID-19 pandemic period (a critical stressful event worldwide) an increase of anxiety and depressive symptoms was observed. The population who reported specific signs to those mental problems augmented cannabis consumption (Bartel et al., 2020) and also other drug use (Roberts et al., 2021). The revision by Gallego-Landin et al. summed up current works concerning the role of the endocannabinoid system regulation in a person's vulnerability to develop depression. Accordingly, stress has negative effects in the organism regardless of the stage in which it is experienced, however the response to stress is not homogeneous, so resilience is an interesting aspect to be studied. Calpe-López et al. proposed, in animal models, that a brief experience of stress in the early life stages increases resilience, attenuating the depression-like behaviors and drug use in stressed animals during later life stages. Moreover, Denny et al. demonstrated, with an artificial intelligence algorithm, individual differences in ethanol consumption (resilience vs. vulnerable) in response to traumatic stress exposure as a consequence of these phenotypes and propose that they might be dependent on modifications in the neuropeptide Y pathway in several amydgalar nuclei.

In addition, anxious symptomatology (related also with mood disorders) is also associated with drug use and there are several aspects (genetics, environment, stress) that can impact this connection. A curious piece of data is how chronotype is linked with psychiatric disorders, with the evening-type individuals suffering more mental health problems (Kivelä et al., 2018). Following that idea, Fernando et al. found that the patterns of daily activity were associated with anxiety levels, with those who presented as late chronotypes presenting with a higher risk for developing the disorder as a consequence of more drugs use, thus offering really interesting new therapeutic approaches to tackle this problem. Furthermore, when a continuous drug consumption is ceased, individuals suffer from withdrawal syndrome, which results in increased anxiety levels, as has been established by Nicolas et al. after intermittent or continuous access to a specific drug (cocaine). Nonetheless, this enhanced anxiety seems unconnected with the incubation of cocaine craving.

Addiction is not only related to mental disorders, but it is also linked with other risks like neurological, cardiovascular, respiratory, and digestive complications. Specifically, cocaine users presented higher risk to develop strokes as a possible consequence of the small vessel pathology developed because of the abused drug when compared with non-users (Shoamanesh et al., 2016). In this Research Topic, Öchsner et al. proposed the analysis of the small vessel diagnostic, which is extremely challenging to detect and measure, by combining fluid-attenuated inversion recovery (FLAIR) scans together with the conventional magnetic resonance technique.

Several therapeutic approaches have been proposed to treat SUDs in their different stages. Some of them are aimed to revert the effects of drugs of abuse, as in the Zhu et al. study, which reported the efficacy of central administration of oxytocin in the medial prefrontal cortex (mPFC) of mice to restore the social avoidance and cognitive impairment following chronic ketamine exposure. Oxytocin infusion in the mPCF was also able to revert, on the one hand, the high levels of inflammatory cytokines evoked by 10-days administration of ketamine to basal and, on the other hand, the ketamine-induced activation of immune markers such as neutrophils and monocytes. Other hormones and peptides have also been suggested to mediate in the effects of drug abuse and to be potential therapeutic targets for SUDs treatment. On this matter, Shevchouk et al. reviewed up-to-date physiological aspects of anorexigenic hormones, such as glucagon-like peptide-1 (GLP-1) and amylin, and orexigenic peptides like ghrelin, all of them best known for their role in appetite regulation, providing evidence that appetite-regulatory peptides modulate reward and addiction processes and deserve to be investigated as potential treatment targets for addiction.

An added interest may have the treatments that mitigate the reinforcing effects of opioids given that, despite being the most effective analgesic drugs in clinical practice, the addictive potential of opioids limits their therapeutic application. Li et al. reported on the essential role of α6β2-containing nicotinic acetylcholine receptors (α6β2^*^ nAChRs; ^*^ designated other subunits) in the mesolimbic dopaminergic neurons for the rewarding effects of morphine. Authors also reported that the central administration of a new antagonist with the highest affinity for α6β2^*^ nAChRs, α-conotoxin TxIB, blocked the acquisition and expression of morphine-induced CPP in mice (as it previously did with the rewarding effects of nicotine, cocaine, and ethanol) without alterations in learning, memory, locomotor activity, and anxiety-like behaviors, and without rewarding effects itself, thus uncovering a new potential target and a drug that might be useful to prevent the abuse of opioids triggered by their clinical use.

Cocaine consumption has gradually increased lately and, in addition, the number of patients seeking treatment for the first time for cocaine use disorders has augmented over the past years in Europe (WHO, 2019). Hence, new strategies aimed to avoid relapses in cocaine-seeking and taking behaviors are vital. One of the triggering factors of these relapses is the retrieval of context memories associated with the drug (Dong et al., 2017). Therefore, it is acknowledged that the study of the underpinning neurobiological mechanisms of drug memory formation will likely help in the development of treatments for relapse prevention (García-Pérez et al., 2016, 2017; Valero et al., 2018). In this regard, Li et al. found that Fyn activity in the hippocampus, through Tau regulation, is essential for the formation of cocaine-associated memory, which in turn is critical for the regulation of cocaine-seeking behavior mediated by cocaine-associated contexts, thus suggesting that Fyn represents a promising therapeutic target for weakening cocaine-related memory and for treating cocaine addiction. Additionally, Ahdoot-Levi et al. published in this collection that dehydroepiandrosterone (DHEA), a neurosteroid that affects brain cell morphology, differentiation, neurotransmission and memory, and reduced drug-seeking behavior in an animal model of cocaine self-administration, has an immediate effect on hippocampal astrocytes density and activation and a continuous beneficial impact on neurogenesis and tissue organization. So, this study emphasizes the requited concert between astrocytes and neurons in the rehabilitation from addiction behavior and propose DHEA as a treatment for correction of brain damage induced by exposure to and abstinence from cocaine.

From what is stated above, it is easy to deduce the relevance of understanding the molecular mechanisms underlying the effects of abused drugs and the risk factors that increase the vulnerability to addiction in order to tailor personal anti-relapse strategies.

## Author contributions

All authors listed have made a substantial, direct, and intellectual contribution to the work and approved it for publication.

## Conflict of interest

The authors declare that the research was conducted in the absence of any commercial or financial relationships that could be construed as a potential conflict of interest.

## Publisher's note

All claims expressed in this article are solely those of the authors and do not necessarily represent those of their affiliated organizations, or those of the publisher, the editors and the reviewers. Any product that may be evaluated in this article, or claim that may be made by its manufacturer, is not guaranteed or endorsed by the publisher.
